# Editorial: Exploring new technologies, investigating new targets, and shedding new light on *Babesia*

**DOI:** 10.3389/fcimb.2022.1004875

**Published:** 2022-09-14

**Authors:** Haiyan Gong, Lan He, Heba F. Alzan

**Affiliations:** ^1^ Key Laboratory of Animal Parasitology of Ministry of Agriculture, Laboratory of Quality and Safety Risk Assessment for Animal Products on Biohazards (Shanghai) of Ministry of Agriculture, Shanghai Veterinary Research Institute, Chinese Academy of Agricultural Sciences, Shanghai, China; ^2^ State Key Laboratory of Agricultural Microbiology, College of Veterinary Medicine, Huazhong Agricultural University, Wuhan, China; ^3^ Department of Veterinary Microbiology and Pathology, College of Veterinary Medicine, Washington State University, Pullman, WA, United States; ^4^ Parasitology and Animal Diseases Department, National Research Center, Giza, Egypt; ^5^ Tick and Tick-Borne Disease Research Unit, National Research Center, Giza, Egypt

**Keywords:** *Babesia*, *in vitro* culture, LDH, TRAP, cytoadhesion

*Babesia* is a tick-borne zoonotic pathogen, found worldwide, that causes significant economic losses in animal husbandry. In order to explore the mechanism involved in tick-*Babesia*-host interactions, as well as potential drug targets or vaccines, we opened the Research Topic “*Babesia*: Biology, Interactions, and Mechanisms of Pathogenesis in Ticks and Its Hosts.” Ultimately, eight articles were accepted for publication.

Wang et al. established a transient and stable genetic manipulation system for *B. duncani* and successfully isolated a single transfected clone. Their work thus provides a genetic modification method suitable for this species, which will greatly facilitate gene function research on this parasite. Meanwhile, Alzan et al. evaluated the impact of an *in vitro* culture system for *B. bovis* and found that long-term *in vitro* culture (> 12 years) led to the loss of the sexual stage–specific 6cysA and 6cysB proteins, resulting in the failure of these parasites to develop sexual forms. Moreover, the adapted *Babesia* in the culture system was smaller in size, less virulent, and unable to be transmitted to cattle *via* ticks. These two articles illustrate that there are two sides to every coin: *In vitro* culture systems can be beneficial for gene functional analysis or drug selection. However, the use of long-term cultured parasites (LTCP) as surrogates of the parental strain to define virulence or vaccine candidates can be disadvantageous, as their genetic composition and phenotype differ from the parental strain. This needs to be properly taken into consideration in upcoming research.

Recently, the essential metabolic pathways that supply adenosine triphosphate (ATP) to *Babesia via* anaerobic glycolysis have begun to attract researchers’ attention as possible drug targets. Lactate dehydrogenase (LDH) is a critical glycolytic enzyme in the anaerobic glycolysis pathway, and has already been suggested as a potential drug target in *Plasmodium*, *Toxoplasma*, *Cryptosporidium*, and piroplasma (Yu et al.). In this Research Topic, the structure of LDH in *B. orientalis* (BoLDH) was solved at a resolution of 2.67-Å, and a comparison to LDH in *B. microti* (BmLDH) was performed (Yu et al.). The results showed that the overall structure of BoLDH and BmLDH is highly conserved, but the catalytic pocket of BoLDH is larger than that of BmLDH. The binding of the active pocket of BoLDH with gossypol was predicted by Discovery Studio software. Further experiments indicated a significant inhibitory effect of gossypol on the *in vitro* growth of *B. bigemina*, suggesting that gossypol may be a potentially effective drug (He et al.).

Merozoite proteins have been described as playing important roles in *B. microti*, yet detailed information on them is still lacking. In the present topic, a thioredoxin (Trx)-like protein was isolated and shown to significantly protect mice from *B. microti* infection (Piao et al.), indicating this protein may be a possible target for *Babesia* control.

To investigate the immune dynamics during *Babesia* co-infection, Zafar et al. infected mice with two species, *B. microti* and *B. rodhaini*. Their results demonstrated a down-regulation of the splenic immune response in acute *Babesia* co-infection, including a significant reduction in splenic B and T cells, and antibody levels, along with a decline in humoral immunity. Infection with *B. microti* affected *B. rodhaini* parasitemia and increased the survival of the co-infected mice. Insights such as these could be useful for developing anti-*Babesia* vaccines in the future.

We also obtained two in-depth reviews on this topic. Paoletta et al. discusses the thrombospondin-related anonymous protein (TRAP) superfamily, which contains either one or two types of adhesive domains (thrombospondin type 1 repeat and von Willebrand factor type A) and is secreted from apical organelles with a micronemal localization. TRAPs have been well studied in *Plasmodium*, and have been proven to be associated with the motility, invasion, and egress of the parasite. With regard to *Babesia*, TRAP-2 from *B. gibsoni* has been shown to bind to erythrocytes. These adhesins from the TRAP- and TRP- families are thus considered attractive targets for developing specific drugs or vaccines.

Allred, meanwhile, has shed new light on *Babesia*’s survival mechanisms. He describes several methods that *Babesia* has evolved to overcome the oxidative environment in the red blood cell. In detail, the *ves* large multigene families in *Babesia* spp. that mediate cytoadhesion have massive sequence variability. Cytoadhesion is thought to prevent infected erythrocytes from passing through the spleen, thus avoiding splenic clearance. Many—perhaps most—VESA1 variants are non-functional in cytoadhesion. However, the extreme environment has stimulated a process of segmental gene conversion that has generated rapid antigenic variation and the selection of VESA1 isoforms capable of binding to one or more endothelial receptors (i.e., cytoadhesion), and thus increased *in vivo* survival of cytoadhesive parasites and immunologic elimination of non-cytoadhesive parasites in the spleen. With repeated rounds of positive selection and amplification, this mechanism could enhance *Babesia*’s chances of survival.

In summary, this Research Topic has presented us with a new genetic manipulation technique, new information on potential vaccine or drug targets, and a unique insight into *Babesia*’s survival mechanism, all of which merit further exploration for the benefit of human and animal health.

## Author contributions

HG organized and wrote the editorial. LH and HA revised it. All authors contributed to the article and approved the submitted version.

## Funding

This work was supported by Shanghai Agriculture applied Technology Development Program China (2020-02-08-00-03-F01485) and the National Key Research and Development Program of China (2017YFC1200202).

## Conflict of interest

The authors declare that the research was conducted in the absence of any commercial or financial relationships that could be construed as a potential conflict of interest.

## Publisher’s note

All claims expressed in this article are solely those of the authors and do not necessarily represent those of their affiliated organizations, or those of the publisher, the editors and the reviewers. Any product that may be evaluated in this article, or claim that may be made by its manufacturer, is not guaranteed or endorsed by the publisher.

